# Society Meetings

**Published:** 1904-10

**Authors:** 


### SOCIETY MEETINGS.

### Buffalo Academy of Medicine.

OFFICERS—SESSIONS, 1904-1905.

President, Arthur W. Hurd; secretary, Edwin A. Bowerman; treasurer, Charles S. Jewett; trustees, Eugene A. Smith, (one year); Ludwig Schroeter, (two years); Thomas F. Dwyer, (three years). The vice-presidents by virtue of being chairman of the various sections are: Allen A. Jones, chairman, section on medicine; Edgar R. McGuire, chairman, section on surgery; William G. Taylor, chairman, section on obstetrics and gynecology; Frederick C. Busch, chairman, section on pathology; ——————————, chairman, section on ophthalmology, otology, etc. The council is therefore composed of the aforementioned officers, the president of the academy of the previous year, J. W. Grosvenor, and the secretaries of the sections, who are: Franklin W. Barrows, secretary, section on medicine; David E. Wheeler, secretary, section on surgery; Francis M. O'Gorman, secretary, section on obstetrics and gynecology; Herman K. DeGroat, secretary, section on pathology; —————— ——————, secretary, section on ophthalmology, otology, etc.

PROGRAM, 1904-1905.

September 6, Surgery—(*a*) Further investigation of the use of hot air in the treatment of surgical infections, Marshall Clinton, (*b*) Cancer of the alimentary canal, with special reference to operations, A. L. Benedict.

September 13, Medicine—(*a*) The use of antistreptococcus serum, Edward Villiaume. (*b*) Asthma and its relations to environment from a blood etiological standpoint, George N. Jack.

September 20, Pathology—Meeting at the Physiological Laboratory, University of Buffalo. (*a*) On the accuracy of the hypobromite method for the determination of urea, F. C. Busch and W. H. Mosher. (*b*) Two cases of bilateral double ureter, James A. Gibson. (*c*) The physiology of the heart with practical demonstrations, F. C. Busch. (*d*) The influence of the Röntgen ray on circulation, Ward Plummer.

September 27, Obstetrics and Gynecology—Sterility as a result of retroversion of the uterus with reports of thirteen cases of pregnancy following Alexander's operation, Herman E. Hayd.

October 4, Surgery—Stated meeting of the academy. (*a*) The question of drainage in operative work on the gallbladder and hepatic ducts, Eugene A. Smith. (*b*) Subject to be announced, John Parmenter.

October 11, Medicine—(*a*) Essential medication in cardiac diseases, Eli H. Long. (*b*) Gastric ulcers, A. L. Benedict.

October 18, Pathology—Subject and place of meeting to be announced.

October 25, Obstetrics and Gynecology—(*a*) Fibroid of the ovary, F. W. McGuire. (*b*) Face presentation, R. L. Banta.

November 1, Surgery—(*a*) Classic art in medicine and surgery, lantern exhibit, Roswell Park. (*b*) The *x*-ray in diagnosis, A. W. Bayliss.

November 8, Medicine—(*a*) The value of certain procedures in the treatment of pneumonia and its complications, DeLancey Rochester. (*b*) The diagnosis of kidney function, Harvey R. Gaylord.

November 15, Pathology—Subject and place of meeting to be announced.

November 22, Obstetrics and Gynecology—Subject to be announced, Matthew D. Mann.

December 6, Surgery—(*a*) Some new views in the treatment of shock, G. W. Crile, Cleveland, O. (*b*) The value of electrical conductivity of the blood and urine in diagnosis of kidney conditions, Harvey R. Gaylord.

December 13, Medicine—Stated meeting of the acadamy. (*a*) Korsakoff's psychosis, Floyd S. Crego. (*b*) The etiology and symptomatology of cerebro spinal meningitis, Frank W. Love. (*c*) Report of a case of meningitis, Ray H. Johnston.

December 20, Pathology—Subject and place of meeting to be announced.

December 27, Obstetrics and Gynecology—Autointoxication and its relation to the pelvic organs, J. E. Walker, Hornellsville, N. Y.

January 3, Surgery—(*a*) Treatment of chancre and chancroid, D. E. Wheeler. (*b*) Subject to be announced, N. W. Wilson.

January 10, Medicine—Subject to be announced, Grover W. Wende.

January 17, Pathology—Stated meeting of the academy. Subject and place of meeting to be announced.

January 24, Obstetrics and Gynecology—An address to empty seats that should be filled by those practising obstetrics, Peter W. Van Peyma.

February 7, Surgery—Subject to be announced, Maurice H. Richardson, Boston, Mass.

February 14, Medicine—Subject to be announced, Jacob S. Otto. The treatment of disease without drugs, A. W. Bayliss.

February 21, Pathology—Subject and place of meeting to be announced.

February 28, Obstetrics and Gynecology—Subject to be announced, Stephen Y. Howell.

March 7, Surgery—Subject to be announced, W. J. Mayo, Rochester, Minn.

March 14, Medicine—(*a*) The care of premature infants, Dewitt H. Sherman. (*b*) Subject to be announced, Albert G. Woehnert.

March 21, Pathology—Subject and place of meeting to be announced.

March 28, Obstetrics and Gynecology—Stated meeting of the academy. (*a*) Cesarian section, Charles E. Congdon. (*b*) Report of a case of Cesarian section, Grosvenor R. Trowbridge.

April 4, Surgery—(*a*) Congenital dislocations of the hip, Bernard Bartow. (*b*) Gangrene of the hollow viscera, Vertner Kenerson.

April 11, Medicine—(*a*) Injuries to the cervical spinal cord, William C. Krauss. (*b*) Subject to be announced, Irving M. Snow.

April 18, Pathology—Subject and place of meeting to be announced.

April 25, Obstetrics and Gynecology—(*a*) Placenta previa, Carlton C. Frederick. (*b*) Abnormal vertex presentation, Earl P. Lathrop.

May 3, Surgery—(*a*) Massage of the heart in cases of impending death, William C. Phelps. (*b*) Arthritis deformans, Prescott LeBreton.

May 9, Medicine—(*a*) Subject to be announced, Albert T. Lytle. (*b*) Subject to be announced, Henry Stadlinger.

May 16, Pathology—Subject and place of meeting to be announced.

May 23, Obstetrics and Gynecology—Alcohol in puerperal sepsis, H. P. Jack, Canisteo, N. Y.

June 13, Annual meeting.

NOTE.—The officers of the sections especially invite the Academy members to present interesting cases and specimens for discussion. This important branch of the work has been neglected in the past, and it is urged that it be given special attention this year, thus adding much to the interest and instructiveness of the meetings.

The Medical Association of Central New York will hold its thirty-seventh annual meeting at Rochester, Tuesday, October 18, 1904, under the presidency of C. A. Vanderbeek, of Rochester. To be included in the program, the titles of papers to be presented should be sent to the secretary by October 1, and to insure prompt publication of all papers in the transactions of the association, the manuscripts should be given to the secretary at the meeting. As these papers are to appear first in the Buffalo Medical Journal, they must not have been published in any other journal, or the secretary cannot undertake to insure their appearance in the transactions. Every member paying the annual dues of one dollar is entitled to a copy of the transactions. Address, C. A. Greenleaf, secretary, 53 South Fitzhugh street, Rochester.

---

The New York and New England Association of Railway Surgeons will hold its fourteenth annual meeting at the Academy of Medicine, New York, November 17-18, 1904, under the presidency of Dr. C. G. J. Finn, of Hempstead, Long Island. Railway officials and all surgeons interested in this work are cordially invited to attend. For particulars address the secretary, Dr. George Chaffee, 338 47th street, Brooklyn.

---

The Association of Military Surgeons of the United States is sending out invitations to the opening exercises of the International Congress of Military Surgeons to be held at the Hall of Congresses, World's Fair, Saint Louis, Mo., on Monday afternoon, October 10, 1904, at 2 o'clock. This is to signal the beginning of the thirteenth session of Association of the Military Surgeons of the United States, as announced in the August edition of the Journal.

For the opening session, to which the general public is invited, the hour of 2 o'clock on Monday afternoon has been fixed, as announced above. The other sessions will convene at the Hall of Congresses at 9 o'clock in the mornings of October 11 to 15—Tuesday to Saturday—inclusive.

Every session will be held in the Hall of Congresses provided by the exposition for the numerous congresses and conventions to be held at Saint Louis during the continuance of the fair. The building is located on the exposition grounds directly west of the Administration Building.

It is announced that the Inside Inn has been selected as hotel headquarters for the meeting. The officers of the association will all be quartered there and mail intended for members of the association should be addressed there. It is important to reserve rooms in advance and blanks for the purpose will be found in the *Journal of the Military Surgeons* for September and October.

Generally—and at all business sessions of the meeting—fatigue or undress uniform will be worn. Full dress uniform or civilian evening dress with the association button or insignia should be worn at evening functions. Officers not now in active service should wear the uniform of the highest grade previously held by them as worn at the time of their withdrawal from active service. For further particulars address the secretary, Major James E. Pilcher, U. S. V., Carlisle, Pa.

---

Dr. Hamilton D. Wey, president of the Medical Society of the State of New York, has appointed the business committee for the next annual meeting which is as follows: Henry Flood, Elmira, chairman; A. Edward Davis, New York; and Leo H. Neuman, Albany. The meeting will be held at Albany, January 31, and February 1 and 2, 1905.

---

The fourth Pan-American Medical Congress will be held in Panama the first week in January, 1905. President Amador of the Republic of Panama has appointed the following-named officers: president, Julio Ycaza; vice-president, Manuel Coroalles; secretary, Jose E. Calvo; treasurer, Pedro de Obarrio, and committee-men, J. W. Ross, J. Tomaselli, M. Gasteazoro.

There will be but four sections: surgery, medicine, hygiene and the specialties, to which the following officers were appointed—namely, surgical section, president, Major Louis LaGarde, secretary, E. B. Herrick; medical section, president, Moritz Stern, secretary, Daniel R. Oduber; section on hygiene, president, Colonel W. C. Gorgas, secretary, Henry E. Carter; section on specialties, president, W. Spratling, secretary, Charles A. Cooke.

---

The Canadian Medical Association held its thirty-seventh annual meeting at Vancouver, B. C., August 23, 24, 25 and 26, under the presidency of Dr. Simon J. Tunstall, of that city, the general secretary being Dr. George Elliott, of Toronto. It was the first time that the association had held a meeting in the Pacific province, and every expectation of a good and a largely attended meeting was fulfilled. The following guests were introduced to the meeting at the opening session: Mr. Mayo Robson, of London, Eng.; Prof. E. C. Dudley, of Chicago; Prof. W. Japp Sinclair, of the Victoria University, Manchester; Dr. McGillivray, of Edinburgh, Scotland; Dr. C. H. Mayo, of Rochester, Minn.; Dr. Oviatt, of Oshkosh, and Dr. Kenneth McKenzie, of Portland, Ore. Mayor McGuigan, of Vancouver, who is a graduate in medicine of McGill University, delivered an address of welcome on behalf of the citizens of Vancouver, and Dr. J. C. Davie, of Victoria, B. C., vice-president of the British Columbia Medical Council, on behalf of the profession of British Columbia.

At the morning session of the second day, Mr. Mayo Robson delivered the address in surgery, accompanied by a lantern demonstration, on the pancreas and the diseases of that organ, which attracted a large audience and commanded great attention.

In the evening the president delivered the annual presidential address, dealing mostly with questions pertaining to the medical politics of the Dominion of Canada. Following the presidential address, Dr. E. C. Dudley gave a very able address in gynecology, illustrated with lantern slides, which proved one of the features of the meeting and was very much appreciated by all present.

The meeting was the third largest in the history of the association, which indicates the great distance to be traveled did not diminish the enthusiasm.

---

The Buffalo Academy of Medicine held meetings during the month of September, 1904, as follows:

*Section on Surgery.*—Tuesday evening, September 6. Program: Status lymphaticus; its relation to sudden death, Edgar R. McGuire. Hydrocephalus, Marshall Clinton.

*Section on Medicine*—Tuesday evening, September 13. Program: The use of antistreptococcus serum, Edward Villiaume. Asthma and its relations to environment from a blood etiological standpoint, George N. Jack.

*Postponed*—The regular meeting of this section, which should, according to the program of session, have been held Tuesday evening, September 20, was postponed.

*Section on Obstetrics*—Tuesday evening, September 27. Program: Sterility as a result of retroversion of the uterus with reports of thirteen cases of pregnancy following Alexander's operation, Herman E. Hayd; discussion by Drs. Mann, Frederick, Howell and Van Peyma.

---

Mississippi Valley Medical Association will hold its 30th annual meeting at Cincinnati, Ohio, October 11, 12, 13, 1904, under the presidency of Dr. Hugh T. Patrick, of Chicago. The headquarters and meeting places will be at the Grand Hotel.

The annual orations will be delivered by Dr. William J. Mayo, of Rochester, Minn., in Surgery, and Dr. C. Travis Drennen, of Hot Springs, Ark., in Medicine.

Request for places upon the program, or information in regard to the meeting, can be had by addressing the secretary, Dr. Henry Enos Tuley, Louisville, Ky., or the assistant secretary, Dr. S. C. Stanton, Masonic Temple, Chicago, Ill.

